# Whiskers as a novel tissue for tracking reproductive and stress-related hormones in North Pacific otariid pinnipeds

**DOI:** 10.1093/conphys/coaa134

**Published:** 2021-01-12

**Authors:** Mandy J Keogh, Patrick Charapata, Brian S Fadely, Tonya Zeppelin, Lorrie Rea, Jason N Waite, Vladimir Burkanov, Chris Marshall, Aubree Jones, Caitlin Sprowls, Matthew J Wooller

**Affiliations:** 1 Division of Wildlife Conservation, Alaska Department of Fish and Game, P.O. Box 110024 Douglas, AK 99811-0024, USA; 2 Division of Wildlife Conservation, Alaska Department of Fish and Game, 1300 College Road, Fairbanks, AK 99701, USA; 3 Biology Department, Baylor University, One Bear Place #97388, Waco, TX 76798, USA; 4 Marine Mammal Laboratory, Alaska Fisheries Science Center, National Marine Fisheries Service, National Oceanic and Atmospheric Administration, 7600 Sand Point Way NE, Seattle, WA 98115, USA; 5 Water and Environmental Research Center, Institute of Northern Engineering, University of Alaska Fairbanks, Fairbanks, AK 99775, USA; 6 Kamchatka Branch of the Pacific Geographical Institute Far East Branch, Russian Academy of Sciences, Petropavlovsk-Kamchatsky 683000, Russia; 7 Alaska Stable Isotope Facility, Water and Environmental Research Center, Institute of Northern Engineering, University of Alaska Fairbanks, Fairbanks, AK 99775, USA; 8 College of Fisheries and Ocean Sciences, University of Alaska Fairbanks, Fairbanks, AK 99775, USA; 9 Department of Marine Biology, Texas A&M University, Galveston Campus, Galveston, TX 77553, USA; 10 Department of Wildlife and Fisheries Sciences, Texas A&M University, College Station, TX 77843, USA

## Abstract

Keratinized tissues, including whiskers, are ideal for acquiring a record of physiological parameters. Most tissues provide a snapshot of physiological status; however, whiskers may support longitudinal sampling for reproductive and stress-related hormones, if hormones are incorporated as whiskers grow and concentrations change with physiological state. Whiskers from female Steller sea lions (*Eumetopias jubatus*) and northern fur seals (*Callorhinus ursinus*) were serially sectioned and pulverized and steroid hormones were extracted. Standard methods were used to validate enzyme immunoassay kits for cortisol, progesterone, 17β-estradiol and testosterone. All hormones were measurable in whisker segments from both species with progesterone concentrations showing cyclical patterns, which appear to signify previous pregnancies or luteal phases. Yearly progesterone concentrations were greater in years a pup was produced compared with years when no pup was observed. Free-ranging female Steller sea lions had reproductive rates between 0 and 1.0 (0.53 ± 0.33, *n* = 12) using a yearly progesterone concentration of 30 pg/mg or greater to classify a reproductive year as producing a pup and below 30 pg/mg as non-reproductive. Cortisol concentrations were greater near the root and rapidly declined, lacking any obvious patterns, throughout the rest of the whisker. Progesterone and testosterone concentrations were able to help determine sex of unknown individuals. Immunohistochemistry revealed that steroid hormones most likely do not leach out of whiskers based on the deposition patterns of progesterone and cortisol being present throughout the whisker length. Overall, measuring steroid hormones in whiskers can reveal individual reproductive histories over multiple years in sea lions and fur seals. Cyclical patterns of δ^15^N were useful for identifying periods of up to ~10 years of growth within whiskers, and measuring both stable isotopes and hormones may be useful for differentiating periods of active gestation from diapause and potentially track multi-year reproductive histories of female otariids.

## Teaser

We measured reproductive and stress-related hormones in whiskers from female northern fur seals and Steller sea lions. Progesterone concentrations displayed cyclical patterns. Stable isotope signatures provided a tool to differentiate years within whiskers and when paired with progesterone concentrations may aid in identifying active gestation or skipped reproductive years.

## Introduction

Two otariid species of conservation concern are found within the North Pacific: the Steller sea lion (*Eumetopias jubatus*) and the northern fur seal (*Callorhinus ursinus*). The western distinct population segment of Steller sea lions is listed as endangered under the Endangered Species Act while the northern fur seal population on the Pribilof Islands is listed as depleted under the Marine Mammal Protection Act. The cause of the continued decline and lack of recovery of both populations remains unknown. Steller sea lions have variable population trends across their distribution, with evidence of lower birth rates at some rookeries contributing to the declining regional trends (Fritz *et al.*, 2014; Altukhov *et al.*, 2015). Altukhov *et al.* (2015) suggested that higher pup survival rate at some sites may be linked to reduced reproductive rates of females. For example, Medny Island and Kozlov Cape rookeries have negative population trends yet high pup survival. While these findings may seem contradictory, the overall decline on these rookeries could be driven by females skipping a year or more between pregnancies, resulting in lower fecundity (Permyakov *et al.*, 2014). The reduced reproductive effort per female would allow increased investment in pups that are produced, ultimately leading to higher survival of pups.

Most otariids are annual breeders with estrus occurring about a week after parturition, with females then undergoing a 3-month delayed implantation (diapause; Pomeroy, 2011; Pitcher and Calkins, 1981; York and Scheffer 1997). Serum progesterone concentrations are similar between pregnant and non-pregnant pinnipeds for months following estrus, preventing the discrimination between pregnant and non-pregnant females until after implantation (Sattler and Polasek, 2017; Shero *et al.*, 2018; Browne et al., 2006). Thus, current methods including field observations of mother–pup pairs (Altukhov *et al.*, 2015) or blood and faecal hormone concentrations (McKenzie *et al.*, 2005) are challenging to implement for determining pregnancy and reproductive rates of otariids. These methods provide a ‘snapshot’ of an animal’s reproductive status, requiring repeated sightings or sampling events, and require a permanent marking for tracking individuals between years. These challenges highlight the need for new methods to assess the reproductive rates of these populations.

Keratinized tissues are increasingly being used to investigate a suite of parameters including steroid hormones (Macbeth *et al.*, 2010; Bryan *et al.*, 2013; Hunt *et al.*, 2014; Keogh *et al.*, 2020a). Longitudinal progesterone profiles along baleen plates reflect known calving events in North Atlantic right whales (*Eubalaena glacialis*; Hunt *et al.*, 2016) demonstrating the potential for continuously growing keratinized tissues to provide reproductive history in free-ranging marine mammals. Whiskers are routinely collected during live-capture research and from bio-sampled pinniped carcasses. Recently, reproductive and stress-related hormones were measured along phocid whiskers (Karpovich *et al.*, 2019; Keogh *et al.*, 2020a). Karpovich *et al.* (2019) reported a rapid decline in cortisol concentrations along phocid whiskers and suggested the pattern may be due to non-linear growth of phocid whiskers, loss or leaching of cortisol or non-keratin tissue within the root of the whisker. Adult phocid seals had greater whisker progesterone concentrations compared with subadults, and for adult harbour seals, concentrations were greater in pregnant or lactating seals while remaining low in non-pregnant females (Keogh *et al.*, 2020a). The use of whiskers would avoid problems associated with a single sample by capturing reproductive and stress-related hormone concentrations sequentially along the length of this continuously growing tissue. Further, whiskers retain carbon and nitrogen isotope signatures that can be used to estimate growth rates (to determine the amount of time the whisker represents) and give timestamps to hormone concentrations (Hirons *et al.*, 2001; Connan *et al.*, 2014; Rea *et al.*, 2015).

**Table 1:** Results of accuracy tests for progesterone, 17β-estradiol, testosterone and cortisol for female Steller sea lions and northern fur seals

	Progesterone	17β-Estradiol	Testosterone	Cortisol
Steller sea lion	y = 0.8447x + 50.742	y = 0.9611x + 18.157	y = 0.9455x + 126.13	y = 0.9934x + 32.908
	R^2^ = 0.972	R^2^ = 0.986	R^2^ = 0.994	R^2^ = 0.977
Northern fur seal	y = 0.8494x—57.991	y = 1.0519x—0.4352	y = 0.9571x + 28.979	y = 0.9904x—111.9
	R^2^ = 0.972	R^2^ = 0.990	R^2^ = 0.999	R^2^ = 0.993

Given the management needs and the challenges faced in estimating reproductive rates in free-ranging pinniped populations, we developed methods for measuring steroid hormones in otariid whiskers. The objectives of this study were the following: (i) to validate enzyme immunoassays (EIAs) used to measure reproductive (progesterone, 17β-estradiol and testosterone) and stress-related (cortisol) steroid hormones in segments of female otariid whiskers, (ii) to investigate the influence of age class and/or reproductive state on whisker hormone concentrations and (iii) to explore if stable isotope signatures are useful for defining the annual cycles along otariid whiskers.

## Materials and methods

### Whisker collection

We used archived whiskers collected from live animals and carcasses as part of ongoing field and captive research programs. The longest whisker for each animal was removed with pliers and stored in a polyethylene sample bag or paper envelope at room temperature until further processing. Steller sea lions with dependent offspring were captured during October 2015 (*n* = 4) at sites in Alaska using remote drug delivery (medetomidine/butorphanol/midazolam) and subsequently maintained under isoflurane gas during sampling and reversed (atipamezole/lantrexone) prior to release (Lander *et al.*, 2020). Three females with dependent offspring were sampled in June 2007 on Lovushki Island, Russia, as described by Waite *et al.* (2012). Whiskers from three female Steller sea lions housed at the Alaska SeaLife Center (ASLC) and two female pups from Chiswell Island (July 2015) were collected under isoflurane gas anaesthesia (Heath *et al.*, 1997) and a fourth female Steller sea lion at ASLC was sampled post-mortem. Whiskers from northern fur seals (*n* = 14) and Steller sea lions (*n* = 7) were also collected post-mortem from commercial fisheries incidental catch or animals found dead on haul-outs and rookeries. In total, 22 female Steller sea lions (2 subadults, 18 adults and 2 pups) and 14 northern fur seals (1 subadult female, 11 adult females and 1 foetus of unknown sex) were included in this study. Age was determined using teeth (Anas, 1970) for three female northern fur seals sampled from fisheries incidental catch and included a 10-year-old, a 4-year-old with a foetus and a 3-year-old. For four Steller sea lions sampled post-mortem, a second whisker was used for immunohistochemistry (IHC) staining for cortisol and progesterone.

### Whisker preparation and sectioning for steroid hormone extraction

Whiskers were sonicated for 10 minutes in deionized water, the outer and inner root sheaths removed when present, then repeatedly cleaned with a Kimwipe® moistened with a 2:1 chloroform:methanol solution to remove surface contaminants and left to dry overnight (Rea *et al.*, 2015). Each whisker was weighed with a microbalance (± 0.1 mg; CP2P Sartorus) and length was measured on a flat surface using a metal ruler (± 0.05 cm) before sectioning. Starting at the proximal end of the whisker (root end), whisker tissue was sectioned using a hand chisel, as previously described for pinniped whiskers (Kernaléguen *et al.*, 2012; Rea *et al.*, 2015). The length of each segment (0.1–0.5 cm) was dependent on the mass of the whisker segments with a target mass of 2.5 mg for northern fur seals and 5 mg for Steller sea lions (Table 1). Using a microbalance (CP2P Sartorus), four to five small pieces were weighed and placed into a 2-mL polypropylene tube (Type I, Sarstedt®). Each sample was pulverized with two 5-mm steel ball bearings at 30 KHz for 12 minutes using a Retsch® MM 400 mixer mill with adapters for 10 vials (Verder Scientific Inc., Newtown, PA).

### Steroid hormone extraction

For extraction, 1 mL of 100% methanol was added to the powdered sample and rotated slowly on a benchtop tube rotator (13 rpm; Thermo Scientific, Radnor, PA) for 24 hours at room temperature (Macbeth *et al.*, 2010; Hunt *et al.*, 2014). Samples were then centrifuged at 10 500 g, 10°C for 13 minutes and the supernatant was transferred to a new polypropylene tube. The remaining pellet was rinsed with 0.2-mL methanol, agitated and centrifuged and the supernatant was combined with the previously removed supernatant. Methanol extracts were stored at ≤−80°C until assayed. Methanol extracts were centrifuged again and a subsample was transferred to a borosilicate glass tube, dried under forced air and reconstituted in an assay buffer specific for each hormone EIA kit.

**Figure 1:**
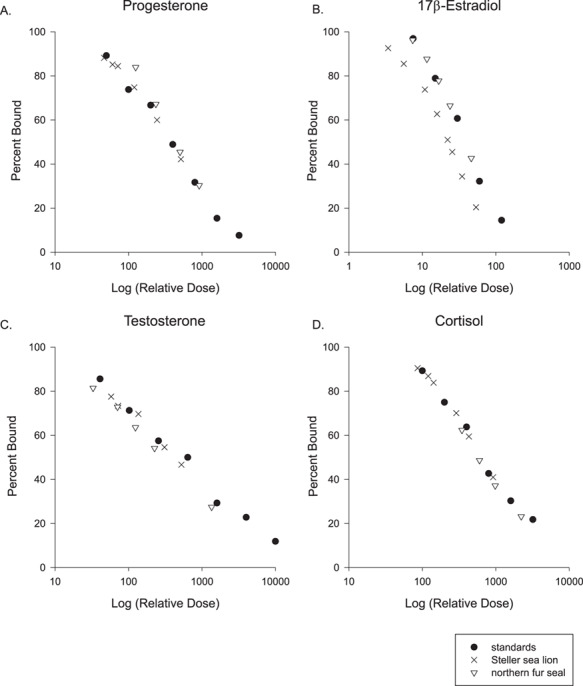
Parallelism results from pooled whisker extracts for females of each species for (**A**) progesterone, (**B**) 17β-estradiol, (**C**) testosterone and (**D**) cortisol

### Hormone EIAs

EIA kits from Arbor Assay (Ann Arbor, MI, USA) were used to quantify the concentration of four steroid hormones: cortisol (K003), progesterone (K025), testosterone (K032) and 17β-estradiol (KB30). These kits were previously used to measure hormone concentrations in phocid whiskers (Keogh *et al.*, 2020a) and otariid fur (Keogh *et al.*, 2020b). Laboratory validations included parallelism, dilution linearity and accuracy (Hunt *et al.*, 2014). Whiskers were processed as described above and methanol extracts were combined from multiple segments and whiskers to make pools for both species. Serially diluted pools were used to determine linearity and parallelism to the standard curves (Hunt *et al.*, 2014). We used ANCOVAs (R version 3.6.1) to assess parallelism and found no difference between the slopes of the serially diluted pools and the standard curves for each EIA kit (Fig. 1; *P* ≥ 0.17). Assay accuracy was assessed by spiking standard curves with an equal volume of a hormone pool (~50% binding from linearity assays for each hormone and species) and assaying alongside standards spiked only with assay buffer. Results were plotted as the observed dose vs. the known standard dose and assessed for linearity, slope and y-intercept. The slope of observed vs. expected concentration was straight with slopes ≥0.972, demonstrating no matrix effect (Table 1). The interassay % coefficients of variation were ≤16% for all assays and the intraassay % coefficients of variation (mean ± standard deviation) were as follows: testosterone (Steller sea lion 2.9 ± 2.6%; northern fur seal 3.4 ± 5.1%), 17β-estradiol (Steller sea lion 4.1 ± 3.5%; northern fur seal 5.3 ± 4.0%), progesterone (Steller sea lion 4.3 ± 3.7%; northern fur seal 4.7 ± 4.1%) and cortisol (Steller sea lion 4.6 ± 3.4%; northern fur seal 5.9 ± 4.4%).

All samples were run in duplicate as per manufacturer’s instructions and all assays included a full standard curve, non-specific binding wells, ‘zero’ (blank) wells and two controls. In most cases, there was enough methanol extract for two hormones to be measured on each segment. Alternatively, methanol extracts from adjacent segments were combined to ensure the sample was above the detection limit of each EIA kit: progesterone (52.9 pg/mL), 17β-estradiol (26.5 pg/mL), testosterone (30.6 pg/mL) and cortisol (45.4 pg/mL). Assay sensitivities were 47.9 pg/mL for progesterone, 2.21 pg/mL for 17β-estradiol, 9.92 pg/mL for testosterone and 17.3 pg/mL for cortisol.

### Immunohistochemistry

We applied IHC methods to a second whisker from four female Steller sea lions to explore the pattern of deposition of progesterone and cortisol. Whiskers were incubated at 37°C in 1.0 × 10^−2^ M Cleland’s reagent for 2–10 days until softened, with stiffer whiskers requiring a longer incubation period. Once softened, the whiskers were removed from the Cleland’s reagent, air dried and then cut into 25-mm segments, starting from the root to the tip. Each segment was then sectioned on a sliding stage microtome with a freezing stage at 7 μm. The whiskers were cut into between 6 and 10 segments with each segment produced between 7 and 20 sections from the microtome.

Individual sections were placed in microcentrifuge tubes with 10× citrate buffer solution (Thermo Fisher Scientific, Waltham, MA, USA). The centrifuge tubes were placed in an autoclave for 15 minutes at high pressure. Once cooled, the samples were removed from the centrifuge tubes and blocked for 30 minutes in 1% bovine serum albumin solution (FisherSci). Sections were rinsed three times in a 0.1-M phosphate buffer solution (PBS) and placed on 1% gelatin-coated slides and allowed to dry and then circled with a PAP pen. Each slide was incubated with 200 μL of primary antibodies, either anti-cortisol or anti-progesterone (Sigma-Aldrich). Two ‘Parafilm® bridges’ were placed on either side of a wax barrier and a coverslip was placed over each slide. Slides were incubated overnight at 2°C in a dark humidity chamber. The slides were then rinsed with PBS for 30 minutes and then again for 5 minutes. Slides were incubated with 200 μL of the secondary antibody (anti-Rabbit IgG; Sigma-Aldrich) in the dark humidity chamber overnight at 2°C with new Parafilm® bridges placed next to the wax barrier. The slides were then rinsed with PBS for 30 and 5 minutes. The 3,3′-diaminobenzidine tetrahydrochloride (Sigma-Aldrich) was pipetted onto the slides for 25 minutes and then rinsed three times with PBS. A counterstain of hematoxylin and eosin (H&E) was used for 45 and 15 seconds, respectively, rinsed with DiH_2_O in between. Following H&E staining, the samples were soaked in 90% glycerin 10% PBS for ~72 hours and then mounted in glycerin onto 5% gelatin-coated slides.

The slides were processed and the best section from each whisker segment was photomicrographed using a SPOT Pursuit camera (Diagnostic Instruments, Sterling Heights, MI) mounted on a Nikon SMZ 1500 stereoscope. The micrographs were scored on a scale of 0–5 by three readers, and the mean score for each section was calculated. Zero represented no staining and 5 represented the highest amount of staining.

### Stable isotopes

To explore tools to define seasons or annual cycles along otariid whiskers, we sectioned whiskers from three adult female Steller sea lions and two adult female northern fur seals and measured stable carbon and nitrogen isotope signatures (expressed as δ^13^C and δ^15^N values) along the whiskers based on the methods reported by Rea *et al.* (2015). We modified our previous method for sectioning whiskers so that every third section was analysed for stable isotope signatures (~1 mm; 0.38 ± 0.07 mg; 0.10–0.50 mg) while two adjacent sections (~2.5 mg each for northern fur seals and ~ 5.0 mg each for Steller sea lions) were processed for progesterone concentrations. This sampling scheme was continued for the entire length of each whisker. The δ^15^N and δ^13^C values for whisker samples were measured using a Costech® Elemental Analyzer coupled to a ThermoFisher Scientific™ Delta V™ Isotope Ratio Mass Spectrometer at the Alaska Stable Isotope Facility at the University of Alaska Fairbanks. Stable isotope ratios are presented in delta (δ) notation:

$$ \delta X=\left(\frac{Rsample}{Rstandard}-1\right)x1000, $$

where Rsample is the ratio of the heavy to light isotope of the sample and Rstandard is the ratio of the heavy to light isotope of the standards (i.e. atmospheric N_2_ for nitrogen and Vienna Pee Dee Belemnite for carbon). The analytical precision was validated by running a laboratory standard (peptone) after every 10 samples, and the standard deviation of these 36 samples was ≤ 0.31‰ for both δ^15^N and δ^13^C values. To explore how the stable isotope values differed near the root and along the length of the whisker, we calculated the atomic carbon to nitrogen ratio (C:N) calculated by the following formula (Nelson *et al.*, 2018):

$$\begin{align*} C:N=\left(\frac{14}{12}\right)x\ \left(\frac{Concentration\ \left(\%\right)\ Carbon}{Concentration\ \left(\%\right)\ Nitrogen}\right). \end{align*}$$

**Table 2:** Mean (± standard deviation) concentrations of hormones (pg/mg whisker), ranges and n (number of whiskers) are reported by species for progesterone, 17β-estradiol, testosterone and cortisol

	Northern fur seal		Steller sea lion	
	Female adults and subadults	1 foetus unknown sex	Female adults and subadults	Two female pups
Progesterone (pg/mg)	114.9 ± 78.9	291.2 ± 162.3	32.3 ± 17.8	44.0 ± 19.1
	(1.4–477)	(171.9–817.5)	(6.1–145.5)	(6.3–69.2)
Whisker segments	1.3 ± 1.2 mg	0.3–0.7 mg	6.4 ± 4.1 mg	1.7 ± 0.5 mg
	0.2 ± 0.2 cm	0.2–0.7 cm	0.5 ± 0.4 cm	0.7 ± 0.6 cm
17β-estradiol (pg/mg)	21.14 ± 10.4		0.4 ± 0.1	
	(7.5–47.1)		(0.2–0.8)	
Whisker segments	9.8 ± 3.6 mg		20.0 ± 5.0 mg	
	1.5 ± 1.0		1.2 ± 0.8 cm	
Testosterone (pg/mg)	4.4 ± 3.0	10.2 ± 13.9	3.3 ± 3.8	
	(0.4–21.6)	(1.2–54.3)	(0.1–21.4)	
Whisker segments	6.6 ± 2.6 mg	0.5 ± 0.1 mg	2.7 ± 1.9 mg	
	0.3 ± 0.3 cm	0.4 ± 0.2 cm	0.5 ± 0.1 cm	
Cortisol (pg/mg)	20.8 ± 29.0		8.0 ± 5.2	
	(6.6–106.9)		(3.1–26.4)	
Whisker segments	29.4 ± 12.4 mg		29.4 ± 7.6 mg	
	1.7 ± 0.9 cm		2.4 ± 1.6 cm	

Following the methods of Rea *et al.* (2015), we measured the distance between adjacent δ^15^N value minima calculated as an annual growth rate, and a monthly growth rate was then calculated by dividing by 12 for both Steller sea lions and northern fur seals.

### Data analysis

Data were analysed with Systat 13 (Systat Software, Inc., Point Richmond, CA). Hormone concentrations are expressed as means plus or minus standard deviation in pg/mg of whisker (herein, pg/mg). All graphs are presented with the x-axis going from the root (proximal) out to tip (distal), so that time is presented backwards. To explore tools to define annual cycles along otariid whiskers, we used previously published estimated whisker growth rates for female Steller sea lions (Rea *et al.*, 2015) and female Subantarctic and Antarctic fur seals (Kernaléguen *et al.*, 2012), since there are not published estimates for northern fur seals. The growth rates were applied to whiskers with known collection dates and we calculated mean yearly progesterone concentrations over one reproductive year (July–June for Steller sea lions; August–July for northern fur seals). For Steller sea lions with known reproductive outcomes in the most recent year, we used a *t*-test to compare progesterone concentrations from females that had a pup with females known to not have been pregnant. We classified the ranges of yearly progesterone concentration as representing a year of producing a pup vs. a non-reproductive year based on samples from females with known reproductive outcomes. Lastly, for five whiskers (three Steller sea lion; two northern fur seal) that were analysed for both progesterone concentrations and stable isotope values, we applied the whisker growth rate for the specific whisker to define reproductive years within that whisker and compared these results to those based on published estimated growth rates.

## Results

Adult female Steller sea lion whiskers were on average longer (25.9 ± 7.8 cm) and heavier (370.4 ± 278.5 mg) than adult northern fur seal whiskers (12.5 ± 3.3 cm; 79.8 ± 23.1 mg). Whiskers became thinner moving towards the tip, requiring the length of segments to increase to ensure the sample masses remained consistent.

### Progesterone

**Figure 2:**
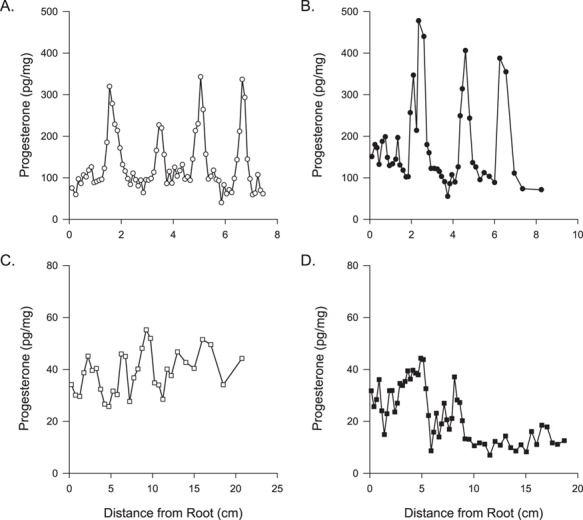
Concentrations of progesterone (pg/mg) along the length of one whisker from two female northern fur seals (**A**, **B**) and two female Steller sea lions (**C**, **D**)

**Figure 3:**
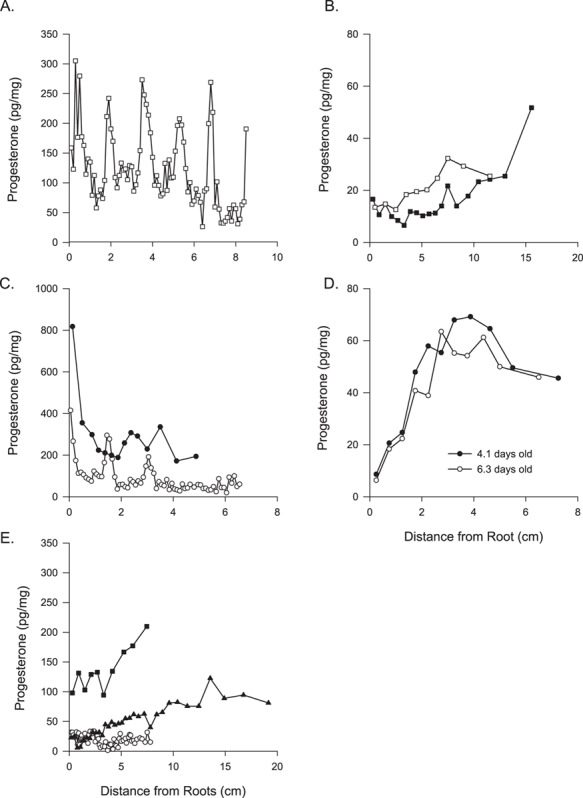
Concentrations of progesterone (pg/mg) along the length of one whisker from (**A**) a 10-year-old female northern fur seal, (**B**) two immature Steller sea lions, (**C**) a 4-year old northern fur seal (○) with a foetus (●), (**D**) two female Steller sea lion pups and (**E**) three northern fur seals with no obvious pattern including a 3-year-old (○).

Progesterone was measurable in whiskers collected from female northern fur seals and Steller sea lions. The progesterone concentrations were greater and the progesterone peaks were more prominent in whiskers collected from northern fur seals compared with Steller sea lions (Table 2; Fig. 2). For both species, whiskers collected from adults had cyclical patterns in progesterone concentrations (Supplemental Figs 1 and 2). Three of the northern fur seals were sampled as fisheries incidental catch and had the ages estimated using teeth: a 10-year-old (Fig. 3A), a 4-year-old with a foetus (Fig. 3C) and a 3-year-old (Fig. 3E). The 10-year-old northern fur seal had six progesterone peaks while the 4-year-old exhibited two full cycles and an increase in progesterone at the root. The 4-year-old female had a foetus present. The foetal whisker was sectioned into 14 segments and displayed a similar pattern in progesterone profile, but with nearly twice the progesterone concentration, as the dam’s whisker (Fig. 3C; Table 1). Whiskers from three northern fur seals, including the incidentally caught 3-year-old, lacked any obvious peaks in progesterone concentrations (Fig. 3E). Similarly, whiskers from two subadult Steller sea lions lacked any cyclical pattern in progesterone with lower concentrations near the root (Fig. 3B). Whereas, the whisker from two Steller sea lion pups of known age (4.1 and 6.3 days old) displayed similar progesterone concentrations and patterns as observed in adult females with the lowest concentrations found near the root (Fig. 3D). The observed patterns in the pup whiskers may also explain the higher progesterone concentrations at the tip of the whiskers found in the subadult Steller sea lions (Fig. 3B). The upsweep in progesterone concentration in the immature female may indicate the tip was grown *in utero*, suggesting nearly all the whisker grown post-parturition is present.

**Figure 4:**
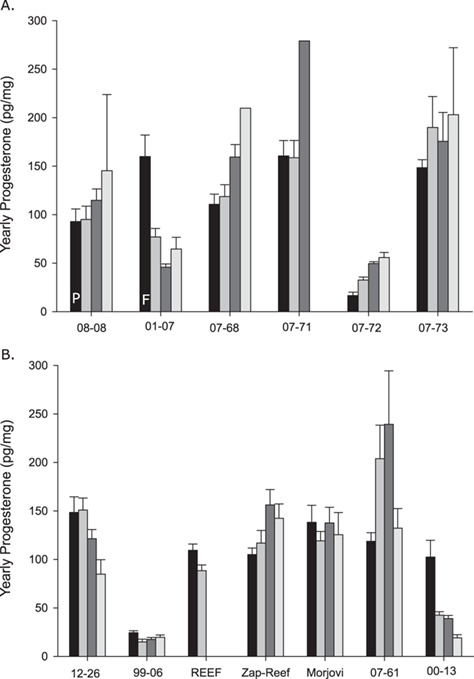
Progesterone concentrations averaged by reproductive year (July–June). Reproductive information was known for two females one with a pup present when sampled (denoted with a P) and a female bycaught in fisheries with a foetus present (denoted with an F). No reproductive information was known for any female fur seal.

Using the collection date, we applied an estimated whisker growth rate of 0.44 cm/mo for Steller sea lions and 0.17 cm/mo for northern fur seals to identified individual years representing approximately one reproductive cycle: July–June for Steller sea lions and August–July for northern fur seals. We then averaged progesterone concentrations within that period to have a mean progesterone concentration for each yearly reproductive cycle within a whisker. For northern fur seals, all whiskers were from free-ranging animals and we had reproductive information on the most recent year from two females (one incidentally caught female with a foetus present when sampled denoted with F and one sampled with a pup present denoted with a P; Fig. 4). There was variability in yearly progesterone concentrations between reproductive years within a whisker as well as between fur seals (Fig. 4).

**Figure 5:**
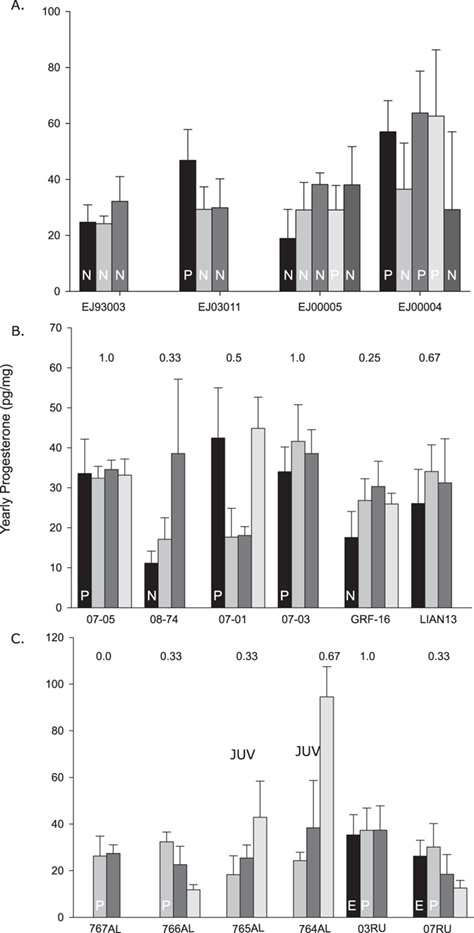
Progesterone concentrations averaged by reproductive year (June–May) in captive Steller sea lions (**A**) and free-ranging female Steller sea lions (**B**, **C**). For reproductive years with known outcome, P denotes years a pup was produced, N for years where no pregnancy occurred (confirmed via necropsy or ultrasound), E for when an embryo was present during necropsy and no letter for years with unknown outcomes. For two females observed nursing a juvenile Steller sea lion prior to sampling, we could not attribute the year the offspring was born, so we note JUV above as it could have been born in the second or third year of the whisker.

**Figure 6:**
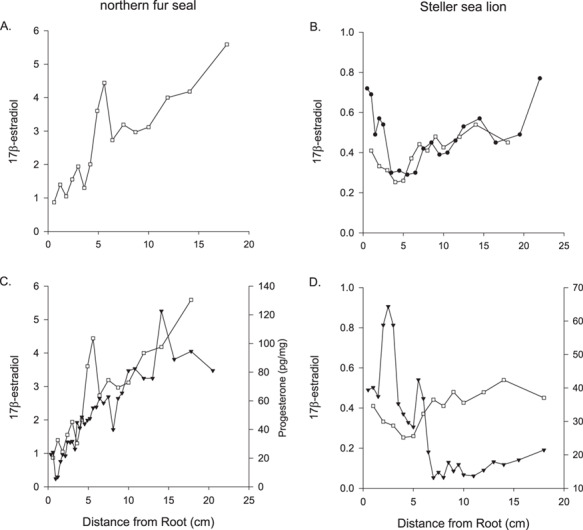
Concentrations of 17β-estradiol along the length of one whisker from (**A**) one northern fur seal and (**B**) two Steller sea lions and the concentrations of progesterone ( ) and 17β-estradiol (□) along one whisker from (**C**) one northern fur seal and (**D**) one Steller sea lion.

For Steller sea lions, we had a reproductive history for multiple years for four adult females under human care at the ASLC with known reproductive histories (Fig. 5A). Three of the sea lions (EJ03011, EJ00005, EJ00004) were participating in a breeding program at the time of sample collection (Sattler and Polasek, 2017; Sattler *et al.*, 2018). The final whisker was collected post-mortem from a 22-year-old female Steller sea lion (EJ93001) and was not part of the breeding program. For free-ranging Steller sea lions, we had known reproductive outcomes in the most recent year with females noted as having a pup present when sampled or a lack of foetus/embryo when necropsied. For each reproductive year with a known outcome, we note P for years when a pup was produced, N for years where no pregnancy occurred (confirmed via necropsy or ultrasound), E for when an embryo was present during necropsy in October and no letter for years with unknown outcomes (Fig. 5). Two free-ranging females were observed nursing a juvenile Steller sea lion, so we note JUV above as it could have been born in the second or third most recent year of the whisker profile (Fig. 5C). In nearly all cases, yearly progesterone concentrations were greater in years with a pup (41.6 ± 14.2 pg/mg, *n* = 11) compared with years with no pup produced (28.0 ± 8.0 pg/mg, *n* = 14). One exception was EJ00005 that had one pregnancy out of 5 years (Fig. 4A). When we compared the most recent reproductive year for each female, so that each female only was sampled once, progesterone concentrations were significantly higher in years that produced a pup compared with years without a pup produced (37.8 ± 10.8 pg/mg, *n* = 8; 21.2 ± 8.4 pg/mg; F_1,11_ = 8.469, *P* = 0.014). If we use a yearly progesterone concentration of 30 pg/mg or greater to classify a reproductive year as producing a pup, and below 30 pg/mg as non-reproductive, we find that individual free-ranging female Steller sea lions across our study had reproductive rates between 0 and 1.0 (0.53 ± 0.33, *n* = 12; Fig. 5B and C). However, we know at least one year was misclassified as non-reproductive when the female was observed with a pup (767AL; Fig. 5C) and a second subadult female appears to have had a pup in Year 3 of whisker growth (08-74; Fig. 5B).

**Figure 7:**
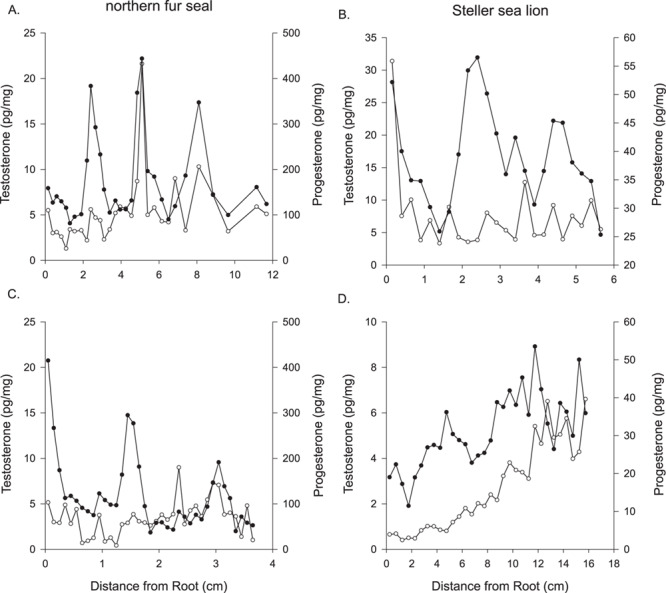
Concentrations of testosterone (○) and progesterone (●) along the length of whiskers from two northern fur seals (**A**, **C**) and two Steller sea lions (**B**, **D**).

**Figure 8:**
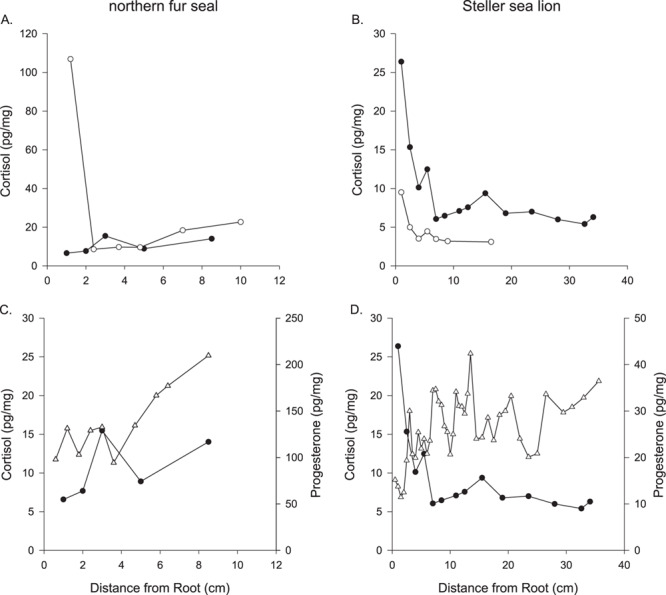
Concentrations of cortisol along the length of two whiskers from (**A**) Steller sea lions and (**B**) northern fur seals. Cortisol (●) and progesterone (Δ) concentrations along one whisker from (**C**) a Steller sea lion and (**D**) a northern fur seal.

### 17β-Estradiol

We combined the methanol extracts from one to three adjacent segments to ensure 17β-estradiol was within the detection level of the kit. The 17β-Estradiol concentrations were higher in northern fur seals compared with Steller sea lions and there was variation in the concentration along the whiskers (Table 2; Fig. 6A and B). In one whisker from a northern fur seal collected post-mortem, there was a general trend of decreasing concentration towards the root of the whisker (Fig. 6A). We measured both progesterone and 17β-estradiol concentrations along one whisker from a northern fur seal and two Steller sea lions. The 17β-estradiol showed variation along the whiskers but there was not an obvious pattern as found with progesterone. In the whisker of one Steller sea lion with two obvious progesterone peaks, the 17β-estradiol concentrations remained fairly consistent, ranging between 0.25 and 0.54 pg/mg (Fig. 6D). In contrast, progesterone and 17β-estradiol in the northern fur seal whisker both had a steep decline in concentration towards the root (most recently grown tissue; Fig. 6C).

### Testosterone

Testosterone concentrations in whiskers were comparable between the northern fur seal and Steller sea lions (Table 2; Supplemental Fig. 3); however, one of the northern fur seal whiskers noted as an adult female had testosterone concentrations much greater than the other females and lacked any progesterone peaks (Fig. 3E; Supplemental Fig. 3A). For all whiskers, we also measured progesterone concentrations and we present the concurrent progesterone and testosterone concentrations from two northern fur seals (Fig. 7A and B) and two Steller sea lions (Fig. 7C and D). Both progesterone and testosterone were measurable in all whiskers demonstrating variability in concentrations. In some cases, progesterone and testosterone displayed similar patterns but not in all cases. As with the progesterone concentrations, the testosterone concentration in the foetal whisker was greater than in the dam’s whisker, but only slightly (Fig. 7C; Supplemental Fig. 3E). There was an elevated testosterone concentration near the root of the foetal whisker (54.3 pg/mg), which was not observed in the maternal whisker (5.1 pg/mg).

### Cortisol

Cortisol was detected in the whiskers of both species with higher concentrations occurring near the root followed by rapid decrease towards the tip of the whiskers (Fig. 8). For one northern fur seal and two Steller sea lions, we measured progesterone and cortisol concentrations in each whisker. Progesterone concentrations in the Steller sea lion whiskers showed variation and cyclical patterns along the length, whereas the cortisol concentration was high near the root followed by a rapid decline in concentrations, though there was some variation in the concentration along the whisker (Fig. 8D). Given the rapid decline observed in cortisol in these whiskers, as well as similar pattern found in phocid whiskers (Karpovich *et al.*, 2019; Keogh *et al.*, 2020a), we limited the number of whiskers in each species used to measure cortisol concentrations.

**Figure 9:**
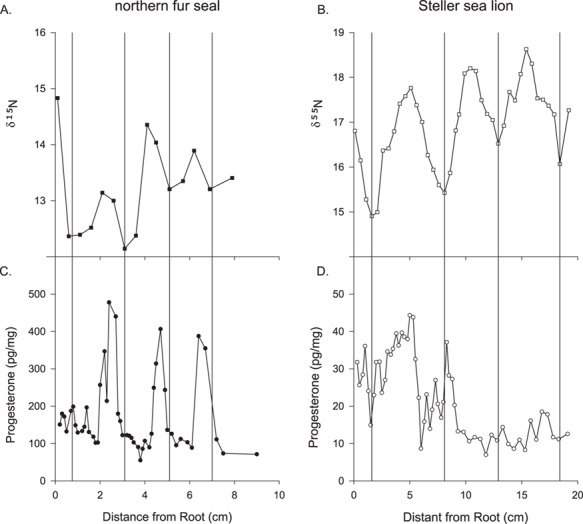
δ15N signatures along the length of a whisker from (**A**) northern fur seal and (**B**) a Steller sea lion. The Steller sea lion was lactating indicating a dependent pup, an embryo was observed during the necropsy. Progesterone concentrations from the same whisker from (**C**) northern fur seal and (**D**) a Steller sea lion. The vertical bars denote the δ15N minimum.

### Stable isotopes

**Figure 10:**
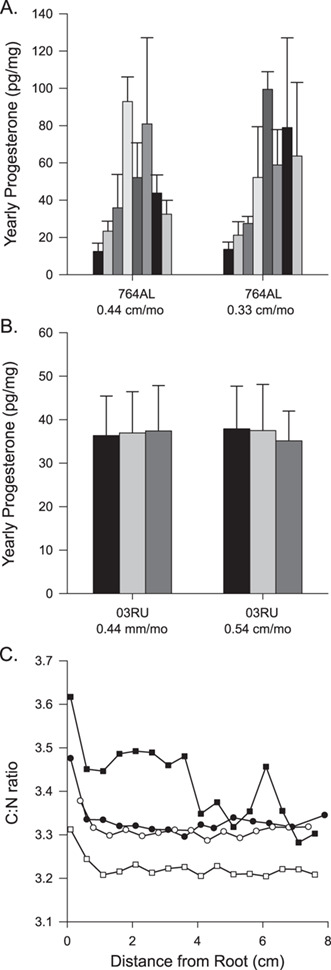
Yearly progesterone concentrations (pg/mg) based on published estimated growth rate (0.44 cm/month) and based on estimated growth rate based on whisker δ15N values from (**A**) Steller sea lion 764 and (**B**) Steller sea lion 03RU and C. Carbon to nitrogen ratios (C:N) along the first 8 cm from whiskers of two northern fur seal (circles) and two Steller sea lions (squares).

We estimated the whisker growth rate based on the δ^15^N value minima to mark 1-year increments along the length of whiskers from two northern fur seals and three Steller sea lions. The results from one northern fur seal and one Steller sea lion are presented in Fig. 9. For the northern fur seal whisker, there were three progesterone cycles present, whereas the most recent growth near the root had consistently low progesterone concentrations, suggesting the female did not ovulate or have a pregnancy in the most recent year (Fig. 9C). Based on the δ^15^N signatures, the two northern fur seal whiskers had growth rates at 0.17 cm/month, the same rate previously estimated for arctic and Antarctic fur seals (Kernaléguen *et al.*, 2012) that we used to estimate yearly reproductive cycles in the northern fur seal whiskers in this study. For the Steller sea lion whiskers, the growth rate based on the δ^15^N signatures that we estimated differed between the three sea lions with growth rates estimated at 0.33, 0.54 and 0.45 cm/month with the whiskers representing 10.8, 5.6 and 3.8 years each. For the Steller sea lion whisker with 3.8 years of growth there was two progesterone cycles preceded by more than 1 year of level concentrations of progesterone (10–18 cm; Fig. 9B and D). When we compared the yearly progesterone concentrations for the two Steller sea lions whose estimate whisker growth rate differed from the published estimates (0.33 cm/month, Fig. 10A; 0.54 cm/month, Fig. 10B), we found that while there was not a dramatic shift in the yearly average of progesterone concentrations, we did find a difference in yearly progesterone concentrations for Years 3–6 for SSL2015 764AL (Fig. 10A).

The stable isotope signatures also provided an opportunity to explore the variability of tissue along the whiskers by looking at the C:N ratios. All four whiskers had an increase in the C:N ratios near the root followed by a plateauing of values (Fig. 10C), suggesting the tissue near the root differs from the rest of the whisker.

### Immunohistochemistry

**Figure 11:**
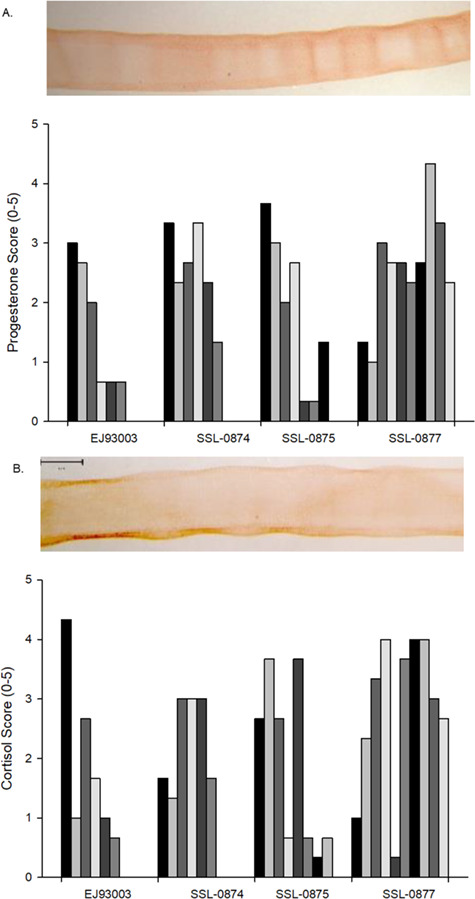
An example of IHC staining in third segment in for SSL2008-75 for (**A**) progesterone and (**B**) cortisol and scoring for (**C**) progesterone and (**D**) cortisol along the length of four female Steller sea lion whiskers.

To explore if the rapid decline in cortisol concentrations was associated with where steroid hormones are deposited in otariid whiskers, we applied IHC methods to one whisker from four female Steller sea lions. All whisker segments had some degree of staining for progesterone and cortisol. Staining for progesterone and cortisol were stronger on the outer portion of the whiskers with progesterone staining also displaying alternating vertical bands of more staining followed by areas of lighter staining through the entire whisker (Fig. 11A and B). This pattern was observed for three of the whiskers, whereas the fourth whisker had more diffuse staining with lighter staining on the outer portion of the whisker in some sections. Cortisol staining showed variability along the length of individual whiskers; however, without the obvious pattern along the length of the whiskers as observed with progesterone.

## Discussion

Steroid hormones were measured throughout the length of all whiskers. Measuring hormone concentrations sequentially along the whisker provided multiple years of steroid hormone concentrations as otariid whiskers are grown and retained over multiple years (Hirons *et al.*, 2001; Connan *et al.*, 2014; Rea *et al.*, 2015; Cherel et al., 2009). Our findings highlight the potential of using whiskers to retrospectively assess the reproductive histories and potential social stressors in female otariids, providing a new tool for researchers and managers.

### Reproductive hormones: 17β-estradiol, progesterone and testosterone

All three reproductive hormones were measurable in whiskers in both species regardless of age class or reproductive status. Progesterone concentrations in whiskers were greater in northern fur seals compared with Steller sea lions (Table 2). This finding was unexpected as circulating concentrations of progesterone in Steller sea lions and northern fur seals during embryonic diapause were comparable (Sattler and Polasek, 2017; Shero *et al.*, 2018). Similar patterns in progesterone along the whiskers were found in both species with multiple peaks of progesterone concentrations along individual whiskers, which we propose indicate previous pregnancies or luteal phases (discussed below).

Similar to progesterone concentrations, northern fur seals had greater 17β-estradiol concentrations and a wider concentration range compared with Steller sea lions (Table 2). Similarly, serum 17β-estradiol concentrations during embryonic diapause were greater with a larger range in northern fur seals compared with Steller sea lions, though the sample size for Steller sea lions was limited (Sattler *et al.*, 2018; Shero *et al.*, 2018). It was not surprising that concentrations of 17β-estradiol in whiskers did not provide a discernable pattern given the length of the whisker segments (a proxy for time) and the highly transient nature of 17β-estradiol. In fact, the peak 17β-estradiol in serum and faeces associated with estrous is often missed (Mellish and Iverson, 2005), even in aquaria or research facilities when samples were collected every 5–7 days (Kiyota *et al.*, 1999; Sattler and Polasek, 2017).

We also measured testosterone, a reproductive hormone increasingly being used to assess social stressors in mammals (Bryan *et al.*, 2013, 2015). Unlike the female reproductive hormones, testosterone concentrations were similar between northern fur seals and Steller sea lions. In some cases, peak testosterone concentrations coincided with the peaks in progesterone concentrations and further research is needed to explore what factors might be influencing the relationship between the concentrations of these hormones in female otariids.

### Reproductive histories

Pinnipeds undergo delayed implantation (embryonic diapause) of several months and non-pregnant females experience pseudopregnancy (Gardiner *et al.*, 1999; Sattler and Polasek, 2017), two states that are not distinguishable with current methods (hormones in serum or faeces). Serum progesterone concentrations in fur seals increased during active gestation, with greater concentrations being reported during late gestation (Daniel Jr, 1975; Boshier, 1981; Katz *et al.*, 2013). Whereas, in Steller and California sea lions (*Zalophus californianus*), progesterone concentrations increased before implantation in both pregnant and non-pregnant sea lions and subsequently declined in non-pregnant females after implantation would have occurred (Greig *et al.*, 2007; Villegas-Amtmann *et al.*, 2012; Sattler and Polasek, 2017).

For both northern fur seals and Steller sea lions, the progesterone concentrations exhibited cyclical patterns along the length of whiskers that we propose are associated with pregnancy or embryonic diapause/pseudopregnancy. We used estimated whisker growth rates to define reproductive years (July–June for Steller sea lions; August–July for northern fur seals) across whiskers and calculated the yearly average progesterone concentrations. Female Steller sea lions that produced a pup had significantly greater yearly progesterone concentrations compared with females that were not pregnant, as confirmed by ultrasound or during necropsy. Based on these findings, we propose using a yearly progesterone concentration of 30 pg/mg for classifying the years that produced a pup. Overall, the reproductive rate of individual female Steller sea lions in our study were lower than we expected (0.53 ± 0.33, *n* = 12), though two females did appear to produce a pup each year for multiple years. The lower reproductive rate in our samples may be, in part, due to the adults sampled in our study. Five of the free-ranging females used to estimate reproductive rates were sampled post-mortem and may have been compromised, which may influence their estimated reproductive rates. Similarly, blubber progesterone concentrations were lower in both alive and dead stranded female California sea lions compared with incidentally caught females suggesting chronic illness or injury influences reproduction in sea lions (Beaulieu-McCoy *et al.*, 2017). Further, studies are needed to assess the rate of misclassifications, as we know we had one misclassification as non-reproductive when a pup was observed with the female. This also contributed to the lower reproductive rate we estimated. While more studies are needed, our findings support the use of whiskers to retrospectively determine the reproductive rates of Steller sea lions, providing a new tool for researchers and managers. Further, the addition of stable isotope signatures to estimate growth rates for individual whiskers may improve our ability to define the reproductive years across whiskers (discussed further below).

We did not have reproductive information for most of the northern fur seals in our study and we, therefore, could not assess differences between progesterone concentrations between years when a pup was produced vs. non-reproductive years. The pattern in progesterone concentrations in northern fur seal whiskers were more prominent and easier to discern than those observed in Steller sea lions (Fig. 2). The utility of using northern fur seal whiskers to investigate reproductive histories is promising and future studies can build upon the methods developed in our study.

Given the variability in whisker growth rates for female Steller sea lions, both previously reported by Rea *et al.* (2015), and in our study, as well as the lack of information on the whisker growth rates in northern fur seals, we propose pairing stable isotope values and progesterone concentrations for each whisker to more accurately define a reproductive year. While our sample size was small, our findings suggest that the variation in estimated growth rates in Steller sea lions may influence when a reproductive year is defined along the whiskers, thereby influencing the yearly progesterone concentrations used to determine in which years a pup was likely produced. In both the northern fur seals and Steller sea lions, the δ^15^N value minima were associated with summer and the whiskers had between 3 and 10.8 years of growth, further highlighting the utility of whiskers to assess reproduction across multiple years from one sample.

Further, the growth rate of whiskers may have contributed to the differences in progesterone and 17β-estradiol concentrations between otariid species, as northern fur seals had a slower estimated whisker growth rate (~0.17 cm/month) compared with the Steller sea lion whiskers (~0.44 cm/month), which may allow more hormones to be incorporated into northern fur seal whiskers during growth. The potential influence of whisker growth rate on hormone concentrations warrants further study before comparisons between species can be made.

### Sexual maturity

Female northern fur seals on average have their first pup at 5–6 years, which may shift over time (York, 1983). Three female northern fur seals had known ages, and as expected, the 3-year-old female was sexually immature with low progesterone concentrations and no obvious peaks (Fig. 3E). The other two females showed multiple peaks in progesterone concentrations suggesting both were sexually mature. In addition to potentially classifying sexual maturity at time of sampling in females, progesterone concentrations may be useful for determining the age of sexual maturity. For example, the 4-year-old northern fur seal had three progesterone peaks, indicating this female was sexually mature at the age of 2 years. Similarly, for one adult Steller sea lion, two progesterone cycles were preceded by low and flat progesterone concentrations near the tip of the whisker, and since this female was observed with a pup, the progesterone cycle nearest the root was associated with active gestation (Fig. 6D). If we apply the estimated growth rate for adult female Steller sea lions (0.44 cm/month; Rea *et al.*, 2015) to this whisker (19.8 cm), we would estimate that the whisker represents 3.8 years, during which two reproductive cycles occurred preceded by nearly two years of immaturity with the minimum age of sexual maturity being 2 years; however, we do not know how much of the whisker was lost due to abrasion leading to an underestimation of ages.

The progesterone concentrations in whiskers from the northern fur seal foetus and Steller sea lion pups support the incorporation of maternal hormones during *in utero* development. The pattern in the progesterone concentrations in the foetal and dam whiskers were similar, with the foetal whisker having greater concentrations and an obvious increase in progesterone near the whisker root with nearly twice the concentration found in the dam. The higher hormone concentration in offspring compared with the dam has been previously reported in fur seals. Meise *et al.* (2016) found that lanugo (natal hair) grown *in utero* similarly had greater concentrations of cortisol compared with fur samples collected from the dam.

The progesterone concentrations in the Steller sea lion pups were surprisingly similar to each other and within the range of concentrations and displaying a similar pattern as found in adult females. Rea *et al.* (2015) suggests *in utero* whisker growth occurs during mid- and late gestation and with both pups being younger than 7 days old when sampled (Maniscalco *et al.*, 2006); nearly all the whisker would have been grown *in utero*. The pattern indicates progesterone concentrations in circulation decrease during late gestation before parturition in Steller sea lions. Sattler and Polasek (2017) also reported progesterone concentrations declined during late gestation in Steller sea lions. Further, we sampled three female Steller sea lions with a newborn pup present and the progesterone concentration in the whiskers had already declined. The whiskers collected from the captive Steller sea lions immediately before parturition also had progesterone concentrations that had already declined. Taken together, our findings support the decline of circulating progesterone before parturition in Steller sea lions. Further, one northern fur seal sampled with a pup present similarly had a decline in progesterone at the δ^15^N value minima (summer), suggesting progesterone concentrations may also decline before parturition in northern fur seals.

### Stress-related hormone: cortisol

Cortisol was measurable in all whiskers; however, concentrations declined rapidly along the whiskers. Karpovich *et al.* (2019) reported a similar pattern in phocid whiskers and suggested the pattern may be due to the non-linear growth of phocid whiskers, loss or leaching of cortisol or non-keratin tissue within the root of the whisker. Our findings provide some insight. First, otariids retain their whiskers for multiple years with a linear growth rate suggesting the decline in cortisol concentrations across pinniped species is likely not due to non-linear growth rates in phocid seals (Hirons *et al.*, 2001; McHuron *et al.*, 2016; McHuron *et al.*, 2020). Secondly, we did not find a similar pattern or any apparent loss of progesterone, 17β-estradiol or testosterone along otariid whiskers. If the decline were due to leaching of cortisol, we would expect a similar effect in the other hormones we measured as they are all non-polar steroids. The IHC staining in Steller sea lion whiskers found progesterone and cortisol were similarly distributed throughout the whisker with greater staining on the outer edge of the whiskers for both hormones. Finally, we ensured that both the inner and outer sheaths were removed from all whiskers, as well as cleaned each whisker to remove external contaminants, and we did not observe any blood within the whiskers (Sergiel *et al.*, 2017). Rea *et al.* (2015) found the isotope signatures near the root were depleted and suggested it was due to additional tissue, beyond keratin, being present in the most proximal portion of the whisker. We found the C:N ratio highest near the root followed by a decline and subsequent plateauing of the C:N ratio, suggesting that the root portion of the whisker includes non-keratin tissue, which may influence the concentration of cortisol, as suggested by Karpovich *et al.* (2019). Studies are needed to better understand how the root portion of the whisker may influence the production and incorporation of cortisol into the whisker and how the cortisol concentrations within the whisker relate to circulating concentrations. Until then, we suggest that cortisol concentrations within whiskers should be interpreted with caution and the proximal 0.5 cm of the whisker (from the root) should not be measured or at least evaluated separately from the cortisol concentrations in the rest of the whisker.

## Conclusions

This study developed methods to measure reproductive and stress-related hormones in the whiskers of two otariid species. For both species, progesterone displayed cyclical patterns likely indicative of previous pregnancies or diapause/pseudopregnancies. For the 10-year-old northern fur seal, six progesterone peaks were present, suggesting at least 60% of the female’s reproductive history incorporated in one whisker. We demonstrated that including stable isotope signatures provides a tool to assign timelines and differentiate among years within otariid whiskers, thereby aiding in discerning years that produced a pup. We suggest a reproductive year with an average progesterone concentration of 30 pg/mg or greater to identify years a pup was produced in Steller sea lions, though a larger study is needed to understand the rate of misclassifications. Cortisol concentrations in otariid whiskers rapidly declined with the highest concentrations being near the root, warranting further research to understand the observed pattern. We found species differences in hormone concentrations, with northern fur seals having greater concentrations of cortisol, progesterone and 17β-estradiol compared with Steller sea lions.

## Funding

This work was supported by the North Pacific Research Board projects 1528 and 1715 awarded to the Alaska Department of Fish and Game.

## Supplementary Material

supplementary_files_coaa134
